# Left ventricular systolic function after inhalation of beta-2 agonists in healthy athletes

**DOI:** 10.1038/s41598-024-74095-z

**Published:** 2024-10-08

**Authors:** H. Persch, D. A. Bizjak, K. Takabayashi, F. Schober, K. Winkert, J. Dreyhaupt, L. C. Harps, P. Diel, M. K. Parr, M. Zügel, J. M. Steinacker

**Affiliations:** 1 Division of Sports and Rehabilitation Medicine, Center of Internal Medicine, Ulm University Hospital, Leimgrubenweg 14, 80975 Ulm, Germany; 2 Hirakata Kohsai Hospital, Hirakata, Osaka Japan; 3 Institute of Epidemiology and Medical Biometry, University of Ulm, Ulm, Germany; 4 Institute of Pharmacy, Pharmaceutical and Medicinal Chemistry, Freie Universität Berlin, Berlin, Germany; 5 Institute for Cardiovascular Research and Sports Medicine, Department of Molecular and Cellular Sports Medicine, German Sports University Cologne, Cologne, Germany

**Keywords:** Beta-2 agonists, Athlete, Sex-specific thresholds, Anti-doping, Echocardiography, Left ventricular function, Global longitudinal strain, Drug discovery, Physiology, Cardiology, Health care, Medical research

## Abstract

Inhaled beta-2 adrenoceptor agonists (iβ2A) are routinely used as bronchodilators in the treatment of asthma. However, their cardiac effects in athletes are scarcely examined. Thus, the aim of this study was to evaluate the effects of iβ2A on left ventricular (LV) systolic function (SF) by echocardiography in healthy, non-asthmatic female and male endurance athletes. A randomized, double-blinded, placebo-controlled, balanced, 4-way complete block cross-over study was conducted. Twenty-four healthy athletes (12f/12m: 22.9 ± 2.7/24.4 ± 4.6 years) randomly completed 4 study arms (placebo; salbutamol; formoterol; formoterol + salbutamol). After inhalation of the study medication, the participants performed a 10-min time trial (TT) on a bicycle ergometer. After each TT an echocardiography was performed to determine LVSF. Blood samples were collected pre, post, 3 h and 24 h post TT. In females, total serum concentrations for salbutamol and formoterol were higher. LV ejection fraction (LVEF) and LV global longitudinal strain (LVendoGLS) showed a treatment effect for the whole study group (*p* < 0.0001) and a sex effect on LVEF (*p* = 0.0085). In women, there was a significant treatment effect for all medication arms (at least *p* ≤ 0.01) both on LVEF and LVendoGLS. In men only formoterol and formoterol + salbutamol displayed a treatment effect on LVEF (*p* = 0.0427, *p* = 0.0330; respectively), whereas on LVendoGLS only formoterol + salbutamol was significant (*p* = 0.0473). The iβ2A significantly influenced LVSF after an acute bout of exercise in healthy endurance athletes. These effects were even more pronounced when combining both iβ2A that supports a dose-dependent effect on cardiac function. Moreover, female athletes had higher serum concentrations of β2 agonists and stronger effects on LVSF compared to male athletes. This is mainly explained by differences in body weight and related plasma volume and may indicate a potential risk when increasing dose above the tested concentrations.

**Trial registration:** At the European Union Drug Regulating Authorities Clinical Trials (Eudra CT) with the number 201,500,559,819 (registered prospectively on 09/12/2015) and at the German register for clinical studies (DRKS number 00010574 registered retrospectively on 16/11/2021).

**Supplementary Information:**

The online version contains supplementary material available at 10.1038/s41598-024-74095-z.

## Introduction

Inhaled beta-2 adrenoceptor agonists (iβ2A) are routinely used as therapeutic drugs in the management of lower airway disease (LAD), which is the most common chronic medical condition found in Olympic athletes^1,2^, with a high prevalence rate especially in endurance and aquatic sports^1–4^. According to current international guidelines the optimal asthmatic drug treatment - depending on symptom level and/or frequency of asthmatic exacerbation - should at least consist of an iβ2A - preferably a long-acting β2A (LABA) such as formoterol - combined with inhaled corticosteroids^5–7^. Short-acting β2A (SABA) such as salbutamol are still used as rescue inhalers/acute symptom relievers^5–8^. Beside their powerful bronchodilatory effect by predominantly relaxing airway smooth muscles via activation of β_2_-adrenoceptors^9–12^, they also seem to have potential performance enhancing, metabolic and anabolic effects that led to extensive research over the last decades^13–23^ and use restrictions by anti-doping regulations in sports^24^. Interestingly, their cardiovascular effects on healthy individuals or athletes have hardly been studied although the potential increase in cardiovascular events when using β2A is well known in pulmonary patients^25–31^ and had significantly influenced the current guidelines^5,6^. Their cardiovascular pharmacologic effects are most likely driven via β_2_ -adrenoceptors, also existing in cardiac muscles (compared to cardiac β_1_ -adrenoceptors in the approximate ratio of 3:7 in ventricles and 1:4 in the atria)^32^, in cardiac adrenergic nerve terminals facilitating release of norepinephrine^33^, and in vascular endothelium as well as skeletal muscle^12,28,30^. This may potentially consequently cause - among others - (reflex-) tachycardia, hypotension with hypoxemia, hypokalemia with increased risk for cardiac ectopies, ventricular tachycardia or fibrillation and/or myocardial infarction^12,28–31^. Arrhythmogenic side effects of β2A are reported, especially with overdoses in sports and their (pro-) arrhythmogenic effect is attributed to their direct β2 stimulant action^11,34^. Snyder et al. found increased plasma norepinephrine levels after administration of nebulized albuterol in healthy individuals with systemic effects such as decreased systemic vascular resistance and increased cardiac output^35^. Syed et al. detected a significant increase in heart rate (HR) after salbutamol nebulization compared to nebulization with normal saline in healthy individuals^36^.

A common doping practice is to combine low doses of different drug formulations (micro-dosing with cocktail formula) to achieve additive/synergistic effects without exceeding the official Word-Anti-Doping Agency (WADA) detection thresholds^37–39^. Regarding the previously described cardiovascular effects, the combination of LABA and SABA might even increase the risk for cardiovascular events. Kalsen et al. had already demonstrated increases in swim ergometer sprint performance and quadriceps maximal voluntary isometric contraction after combined inhalation of salbutamol, formoterol and salmeterol in elite swimmers with (n = 13) and without LAD (n = 17)^40^. However, the study group did not investigate the influence on cardiac/cardiovascular effects. Moreover, little is known about possible different sex-specific effects of iβ2A on athletes^8^.

Therefore, the aim of this study was to examine whether the single use of inhaled salbutamol and formoterol as well as their combined intake at WADA-permitted doses might affect left ventricular (LV) systolic function (SF) measured non-invasively by 2-dimensional (2D) transthoracic echocardiography (TTE) and speckle tracking imaging (STE) in healthy, non-asthmatic female and male endurance athletes after an acute bout of exercise.

## Methods

This prospective randomized, double-blinded, placebo-controlled, balanced four-way cross-over phase I human pharmacological trial was conducted at the Division of Sports and Rehabilitation Medicine at the University Hospital Ulm, Germany. The study was approved by the ethics committee of Ulm University, Germany (reference number 64/19) and was performed in accordance with the provisions of the Declaration of Helsinki as revised in 2013, with the International Conference on Harmonization of Good Clinical Practice. The study was registered at the European Union Drug Regulating Authorities Clinical Trials (Eudra CT) with the number: 2015-005598-19 (registered prospectively on 09/12/2015) and at the German register for clinical studies (DRKS number 00010574; registered retrospectively on 16/11/2021). All participants were informed about possible risks and gave their written informed consent before any study-related treatment or examination could take place.

### Sample size and study design

For a balanced 4-way complete block cross-over design there is a solution involving 4 sequences, which minimizes the chance and effects of first order carry over effects. For each sex, 3 replications of each line were possible to use in this Phase I trial, resulting in 12 participants for each sex and a total of 24 participants. This is a feasible number of participants for phase-I-trials. Three replications of each situation give the statistical potential of estimation of the variance which is needed in mixed model analysis. By neglecting carry-over effects by the procedure of Gaus and Högel^41^ there are 12 observations for each of the four treatment-combinations expecting enough power for investigation of differences.

**Fig. 1:**
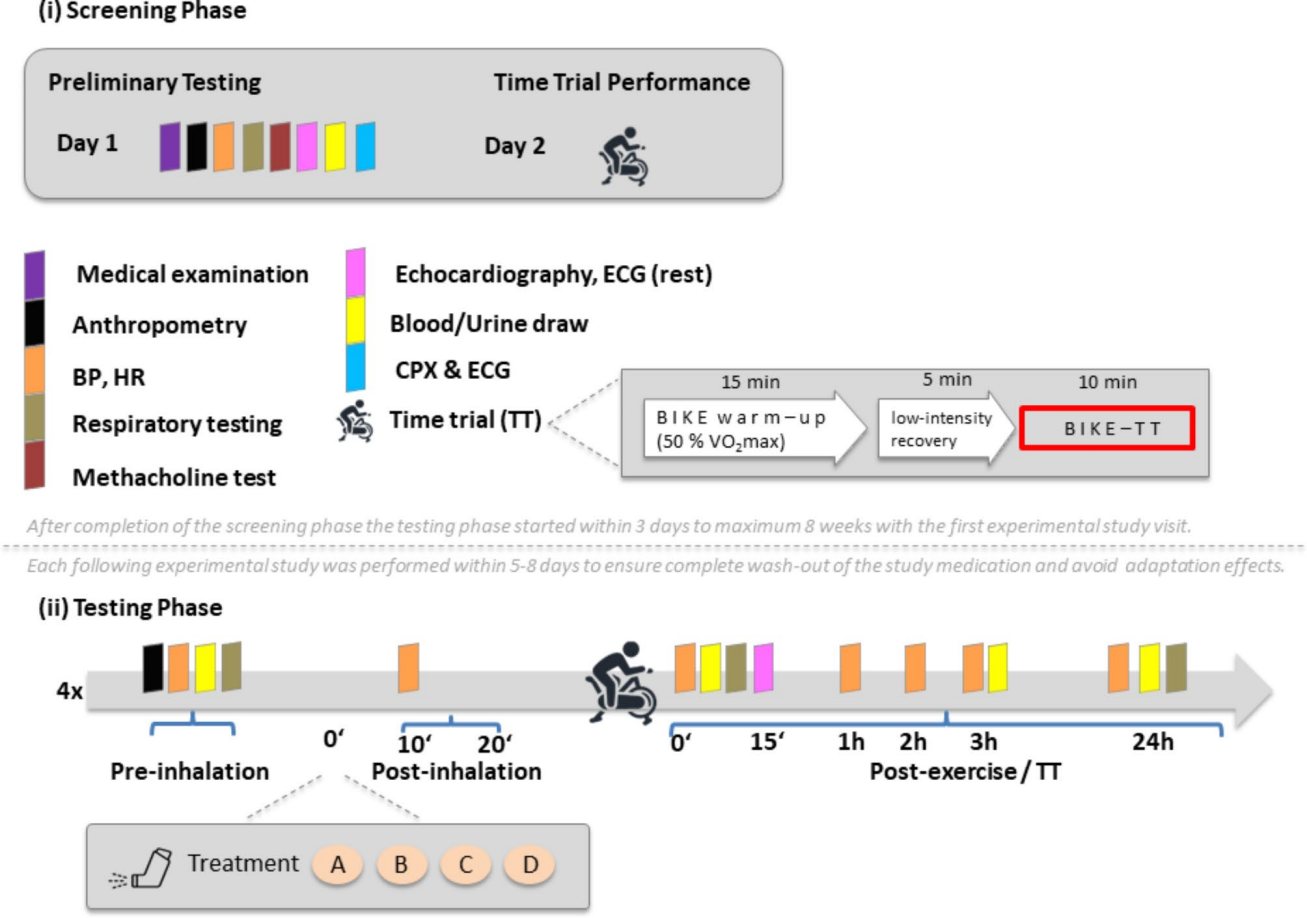
Schematic illustration of the work- and time-flow of the study conduct consisting of a (**i**) screening and a (**ii**) testing phase with four experimental study visits for each respective study arm; modified from Zuegel et al.^42^. (i) After the initial screening phase with preliminary testing and screening for inclusion and exclusion criteria on Day 1 and familiarization with the time trial (TT) on Day 2, eligible participants were randomized to four possible sequences by a stratified block randomization according to sex. Each TT consisted of a 15 min bike warm-up at 50% of participants` individual V̇O_2_max, followed by a 5 min low-intensity recovery exercise and ended with an "all-out" TT over 10 min, where participants aimed to reach the highest average output possibile within 10 min at an even pace starting with a workload equaling to 90–95% of their previously determined Pmax (during cardiopulmonary exercise test (CPX) at screening phase on Day 1) individually increasing their workload by increasing their cadence. All TT were performed on the same cycle ergometer as during the CPX and participants` specific settings (saddle and hand bar position) were the same during each experimental study visit. (ii) The testing phase with the first experimental study visit had to start within 3 days to maximum 8 weeks after completion of the screening phase and each following experimental study visit had to be performed within 5–8 days in order to ensure complete wash-out of the study medication and avoiding adaptation to the exercise protocol. During the testing phase participants were asked to avoid any exhausting activity and alcohol intake 24 h before each experimental study visit and should be fasting at least 8 h prior to TT. All experimental study visits took place at the same time of the day in order to minimize the influence of circadian rhythm variations. At each of the four experimental study visits initially body core temperature was measuered, followed by anthropometry, blood pressure (BP), heart rate (HR) at rest, blood and urine collection including a urine pregnancy test in female participants in order to exclude pregnancy before intake of the study medication. Afterwards all participants were provided with a defined and standardized meal in order to standardize nutritional status. Ten min after inhalation to the study medication BP and HR was measured again. Twenty 20 min after inhalation of the study medication the TT started. After cessation of each TT (1) BP and HR where measured after approximately 5 min, 1 h, 2 h, 3 h and 24 h. (2) blood and urinary draw were performed 5 (−10) min, 3 h and 24 h and (3) an echocardiography was done at the latest 15 min after TT cessation. Participants` adherence to the study protocol was regularly checked and recorded.* A* study arm /treatment A with placebo,* B* study arm/treament B with 1200 µg salbutamol,* C* study arm/treatment C with 36 µg formoterol,* D* study arm/treatment D with the combined application of 1200 µg salbutamol + 36 µg formoterol,* BP* blood pressure,* HR* heart rate,* ECG* 12-lead electrocardiogram,* CPX* cardiopulmonary exercise test,* TT* time trial,*VO*_*2max*_ maximum oxygen uptake. Symbols of the testing phase are placed according to the chronological order of the tests and examinations.

The detailed study design and protocol has already been published^42^. In brief, the study consisted of a (1) screening (two days) and (2) testing phase with four experimental visits (each over two days) for respective study arms (Fig. 1).

1. The screening phase included preliminary physiological and performance testing in order to determine study participants eligibility and physical fitness. On day 1 individual medical history was assessed with detailed medical and physical examination including measurements of anthropometry, blood pressure (BP), heart rate (HR), respiratory and methacholine testing as bronchial challenge test to reliably exclude LAD, 12-lead electrocardiogram (ECG) and 2D TTE at rest, laboratory testing including blood and urine draw and a cardiopulmonary exercise test (CPX) on an electronically braked cycle ergometer (Lode Excalibur Sport, Groningen, The Netherlands) for evaluation of participants` V̇O_2_max (as one main inclusion criteria). The CPX included a (1) continuous incremental test (starting with a workload of 25 W and mechanical output increased 20–35 W/min in female or 35–45 W/min in male participants, respectively (depending on individual fitness and body mass), leading to voluntary exhaustion or test termination when cycling cadence was < 60 rpm after 10–12 min; the highest V̇O_2_was averaged over 30 s and temporarily excepted as V̇O_2_max and power at V̇O_2_ max (Pmax) was calculated during the same interval). After a 15 min break a (2) verification test (a supra-maximal constant-load test with 110% of Pmax lasting approximately 5 min in order to verify or correct the temporary excepted V̇O_2_max) was performed. On day 2 a test time trial (TT) was conducted on the same cycle ergometer as during the CPX allowing participants to get familiarized to the exercise test before performing the TT during the four experimental study visits in the testing phase.

All eligible participants were randomized to four possible sequences by a stratified block randomization according to sex after completion of the screening phase and had to enter the testing phase within a predefined time slot (s. Fig. 1).

(2) The testing phase as well as the TT are illustrated and described in Fig. 1. In short, the randomized participants had to completed all 4 study arms, i.e., periods with the inhaled treatment A: placebo + placebo; B: salbutamol (cumulative dose: 1200 µg) + placebo; C: formoterol (cumulative dose: 36 µg) + placebo; D: combination of salbutamol + formoterol (cumulative dose: 1200 µg salbutamol + 36 µg formoterol). The used doses were higher than the usual prescribed for pulmonary patients, but lower than the current WADA-allowed thresholds. Due to authority restrictions by the German Federal Institute of Drugs and Medical Devices the maximum allowed administered cumulative iβ2A dosage in this study was for salbutamol 1200 µg and for formoterol “only” 36 µg (as the study was classified as a phase I human pharmacological trial), although the current allowed-WADA thresholds in 24 h are 1600 and 54 µg, respectively^24^. Thus, for each treatment a medication test kit was prepared in the central pharmacy at University Hospital Ulm and consisted of six blinded spray inhalers, which were medication and/or placebo (i.e. four blinded medication kits per participant containing 24 sprays in total) in order to ensure double-blinding.

For safety reasons, the investigator received a set of sealed envelopes, one for each randomization number, including the treatment sequence. These envelopes could have ben opened in emergency situations (such as cardiac rhythm disorder or syncope).

Twenty minutes after inhalation of the study medication the TT was performed. As outcome variables serum concentration of the iβ2A pre, post, 3 h post and 24 h post TT were measured and a 2D TTE was done for each study arm to determine LVSF at the latest 15 min post exercise.

All participants were regularly asked to report any side effect, adverse event (AE) and/or adverse drug reaction (ADR) and if present the AE/ADR was documented in the safety case report form (CRF). The safety assessment (grading of severity; decision on serious adverse event (SAE), serious unexpected adverse reaction (SUSAR) and expected serious adverse reaction (SAR)) and the reporting were standardized and a Data and Safety Monitoring Board (DSMB) was additionally established to ensure participants` safety.

### Study population

Participants were healthy competitive athletes aged 18 to 45 years and endurance trained with a V̇O_2_max ≥ 52 ml/kg/min in male or ≥ 42 ml/kg/min in female athletes, respectively (V̇O_2_max was measured and verified on a bicycle ergometer during screening phase; s. Fig. 1). The health status as well as the physical function was ascertained during a thorough preliminary testing (Fig. 1). Study inclusion was only possible with a personally signed and dated consent form and if none of the stringent exclusion criteria were present. Exclusion criteria included (1) women with a positive pregnancy test on enrolment and prior to each study medication administration, (2) allergy/hypersensitivity, (3) a positive methacholine challenge test, (4) contraindication or (serious) adverse reaction of any component of the study medication, (5) adverse medical history and concurrent disease, and (6) subjects who were incapable of giving informed consent. Further details on exclusion criteria can be found in the published study protocol^42^.

### Echocardiographic assessment and outcome

All participants underwent a routine 2D TTE including Doppler- and STE using an EPIC7 cardiology ultrasound system with a x5-1 transducer (Philips Healthcare, Andovar, Massachusetts) during the preliminary screening and 15 min after termination of each TT. For optimal views the participants were placed in the steep left-lateral decubitus position with raised left arm and a special focus was put on optimal image acquisition (avoiding foreshortening of the LV). All acquired datasets were exported and digitally stored for off-line image analysis. Echocardiographic parameters were measured according to current international guidelines^43^. For strain analysis, images had to be acquired with at least 40 frames per second and were analysed off-line with a dedicated software (TomTec Image Arena Version IA4.6.4 TTA2.20.01, TomTec Imaging Systems GmbH, Unterschleissheim, Germany). Quantification and analyses were performed by two cardiologists experienced in TTE, blinded to treatment and according to current international recommendations^43–45^.

### Measurement of β2 agonist concentrations and heart rate

Serum concentrations of salbutamol and formoterol were measured by ultra-high-performance liquid chromatography hyphenated to tandem mass spectrometry (UHPLC-MS/MS). For serum analysis, solid-phase-extraction for sample preparation was used. Method details are available in Harps et al.^46^.

During the testing phase HR was measured at predefined time points using a heart rate monitor (s. Fig. 1).

### Statistical analysis

Detailed statistical analysis procedure can be found in the study protocol and in the statistical analysis plan^42^. A mixed effects regression model approach was applied to analyse each endpoint. This approach included period effects (differences within the study arms 1–4, which may indicate e.g. a training effect by adaptation to the exercise protocol or no complete wash-out of the study medication), treatment (by the study medication itself) and sex. Additionally, similar mixed effects regression models were fitted separately for sex to investigate the stability of the results from the main analysis. All statistical tests performed were two-sided at a significance level of 5%. Because of the explorative nature, no adjustment for multiple testing was done. All results from the statistical tests are regarded as hypothesis generating only, and not as proof of efficacy. The endpoints were evaluated with a full intention to treat. Missing values in measurements were incorporated in the analysis by using mixed models. All statistical analyses were performed using SAS, version 9.4 (SAS Inc. Cary/NC, USA) under Windows 10. For determining serum concentration differences of iβ2A between each sex, a 2-way ANOVA with following Tukey`s multiple comparisons test was conducted with GraphPad Prism 9.5 (San Diego, CA, USA). Scatter plots and Spearmans rank correlation coefficients were used to investigate the association between HR and the serum sample concentrations of salbutamol and formoterol. GraphPad Prism 9.5 was used for graphical representation, the supplementary figures S2A-S2C were created with R (version 4.3.2). Intra-observer variability was determined by having the observer who measured the data in all participants re-measure the LVSF in 10 echocardiographic datasets more than one year apart from the primary measurement. Inter-observer variability for LVSF was determined by a second independent blinded observer who measured these variables in 10 randomly selected datasets. Observer variabilities were assessed using intraclass correlation coefficient (ICC). ICC estimates and their 95% confident intervals (CI) were calculated based on a mixed-effects model.

## Results

### Participant enrollment, characteristics and echocardiographic parameters

**Fig. 2:**
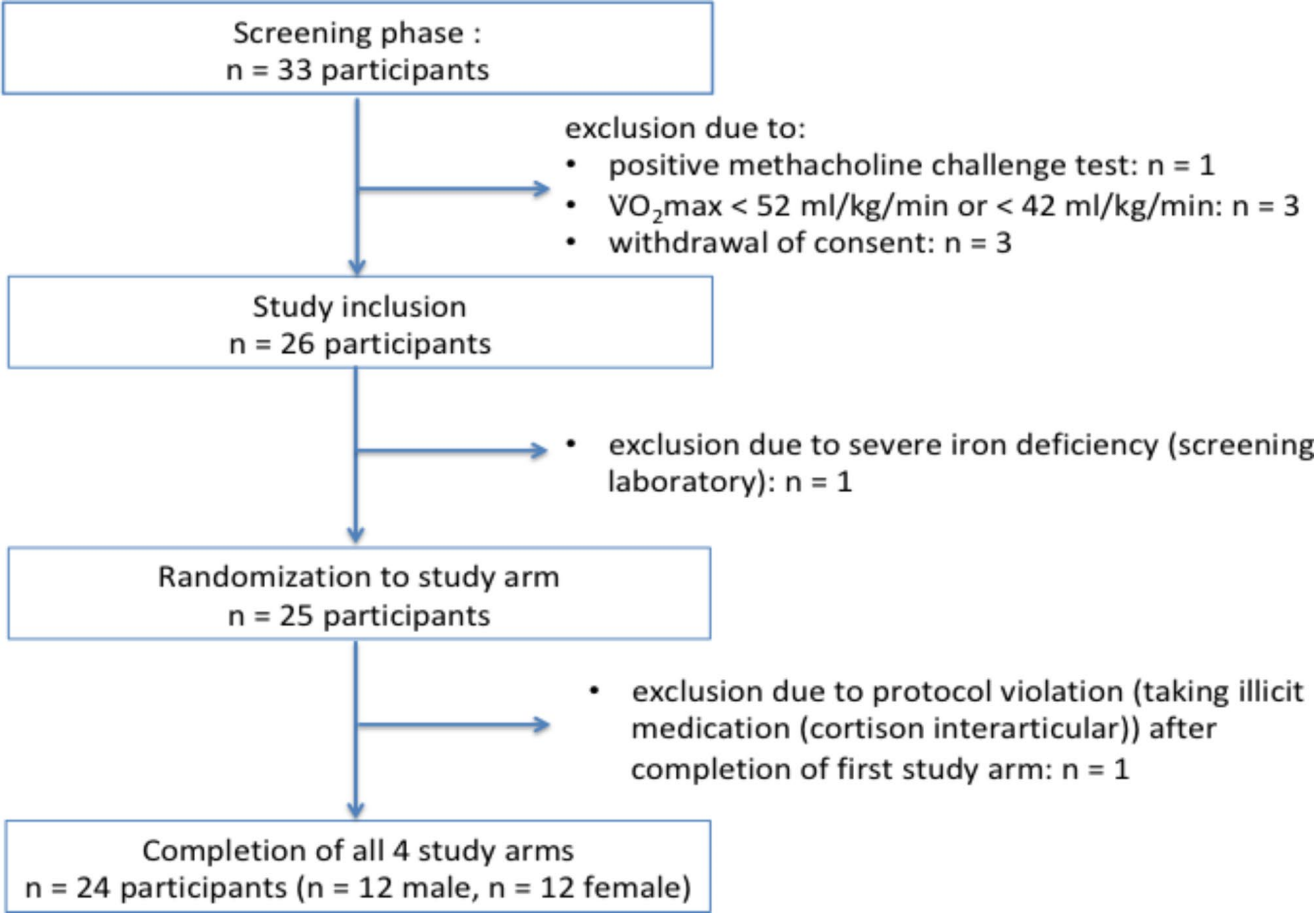
Flow chart of participant enrolment.

**Fig. 3:**
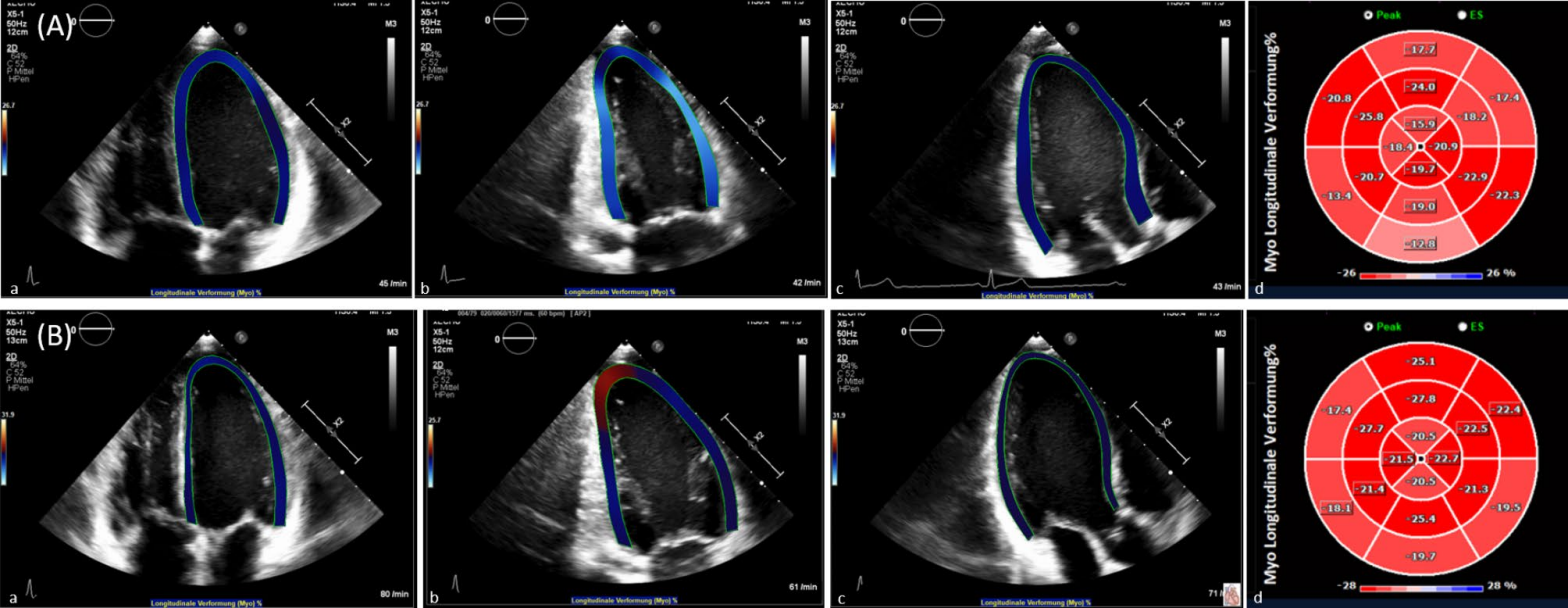
Representative composite of a left ventricular global longitudinal strain (GLS) analyses using TomTec software. (**A**): at baseline (a) apical 4-chamber view, (b) apical 2-chamber view, (c) apical long-axis view, and (d) corresponding bull`s eye view (16 segment model) for peak systolic strain with endoGLS − 20.48%. (**B**): 15 min after Time Trial Performance (a) apical 4-chamber view, (b) apical 2-chamber view, (c) apical long-axis view, and (d) corresponding bull`s eye view (16 segment model) for peak systolic strain with endoGLS −23.48%.

Potential participants were asked about their endurance sport experience and recent competition race results, thus creating a ranking-list. The highest-ranked 33 competitive cyclists, triathletes, middle and long distance runners were finally selected for the screening phase. Twenty-five individuals met the necessary inclusion and none of the exclusion criteria and were randomly assigned to study arms; one subject dropped out due to protocol violation after completion of the first study arm (Fig. 2). Therefore, twenty-four participants completed all study arms. The whole study population was Western European Ancestry with a median age of 23 ± 3 years in female and 25 ± 5 years in male participants (Table 1; for pulmonary function test please s. Supplementary Table S1). Echocardiographic parameters at screening are presented in Table 2 with normal findings and good LVSF in all participants. All echocardiographic datasets displayed sufficient image quality for quantification and strain analyses (Fig. 3).

**Table 1:** Participant demographics and clinical characteristics.

	Female (*n* = 12)	Male (*n* = 12)
Age, mean (years)	23 ± 3	25 ± 5
Height (cm)	170.8 ± 5.8	180.5 ± 3.9
Weight (kg)	61.9 ± 5.7	73.8 ± 5.4
BMI (kg/m^2^)	21.2 ± 1.8	22.7 ± 1.8
BSA (m^2^)	1.72 ± 0.10	1.93 ± 0.07
HR (bpm)	59 ± 12	58 ± 14
Systolic BP (mmHg)	110 ± 9	115 ± 10
Diastolic BP (mmHg)	64 ± 7	75 ± 8
Preliminary V̇O_2_max (l/min) (incremental test)	3.39 ± 0.39	4.74 ± 0.61
Verified V̇O_2_max (l/min)	3.37 ± 0.37	4.71 ± 0.57
Calculated verified VO_2_max (ml/min/kg)	54.8 ± 6.8	63.7 ± 5.7
PP at V̇O_2_max (W) (incremental test)	304 ± 29	418 ± 26
110% of PP (W) (verification test)	336 ± 35	459 ± 29
HR at V̇O_2_max (bpm)	185 ± 9	186 ± 12

Data are given as mean ± standard deviation or absolute numbers, unless otherwise stated.

*BMI* body mass index,* BSA* body surface area by Du Bois,* HR* heart rate,* BP* blood pressure,*VO*_*2max*_ maximum oxygen capacity,* PP* peak power.

**Table 2:** Echocardiographic parameters.

	Female (*n* = 12)	Male (*n* = 12)
iVSd (mm)	9 ± 1	10 ± 1
LVPWd (mm)	8 ± 1	9 ± 1
LVESD (mm)	31 ± 4	34 ± 3
LVEDD (mm)	49 ± 4	54 ± 3
LVESV (ml)*	51.1 ± 14.7	62.8 ± 14.6
LVESV/BSA (ml/m^2^)	29.5 ± 7.6	32.5 ± 7.3
LVEDV (ml)*	122.3 ± 20.4	145.0 ± 22.6
LVEDV/BSA (ml/m^2^)	70.9 ± 10.3	75.1 ± 10.9
EF biplane (%)*	58.5 ± 6.6	56.9 ± 5.9
Mitral E (cm/s)	82 ± 10	85 ± 12
Mitral A (cm/s)	49 ± 10	51 ± 11
Mitral E/A	1.70 ± 0.24	1.75 ± 0.48
Mitral septal e`(cm/s)	12 ± 1	13 ± 2
Mitral lateral e`(cm/s)	17 ± 3	18 ± 3
Mitral average E/e`	5.64 ± 0.48	5.69 ± 1.01
LV endocardial GLS (%)*	− 21.9 ± 3.5	− 22.1 ± 3.1
LV myocardial GLS (%)*	− 19.0 ± 2.5	− 18.9 ± 3.0
LV GRS*	49.4 ± 13.2	47.9 ± 11.7

Data are given as mean ± standard deviation, unless otherwise stated.

*iVSd* interventricular septal wall thickness at end-diastole,* LV* left ventricular,* PWD* posterior wall thickness at end-diastole,* ESD* end-systolic diameter,* EDD* end-diastolic diameter,* ESV* end-systolic volume,* EDV* end-diastolic volume,* BSA* body surface area by Du Bois,* EF* ejection fraction,* GLS* global longitudinal strain,* GRS* global radial strain.

All parameters with * were analyzed with TomTec Software.

### Changes in LVSF

**Fig. 4:**
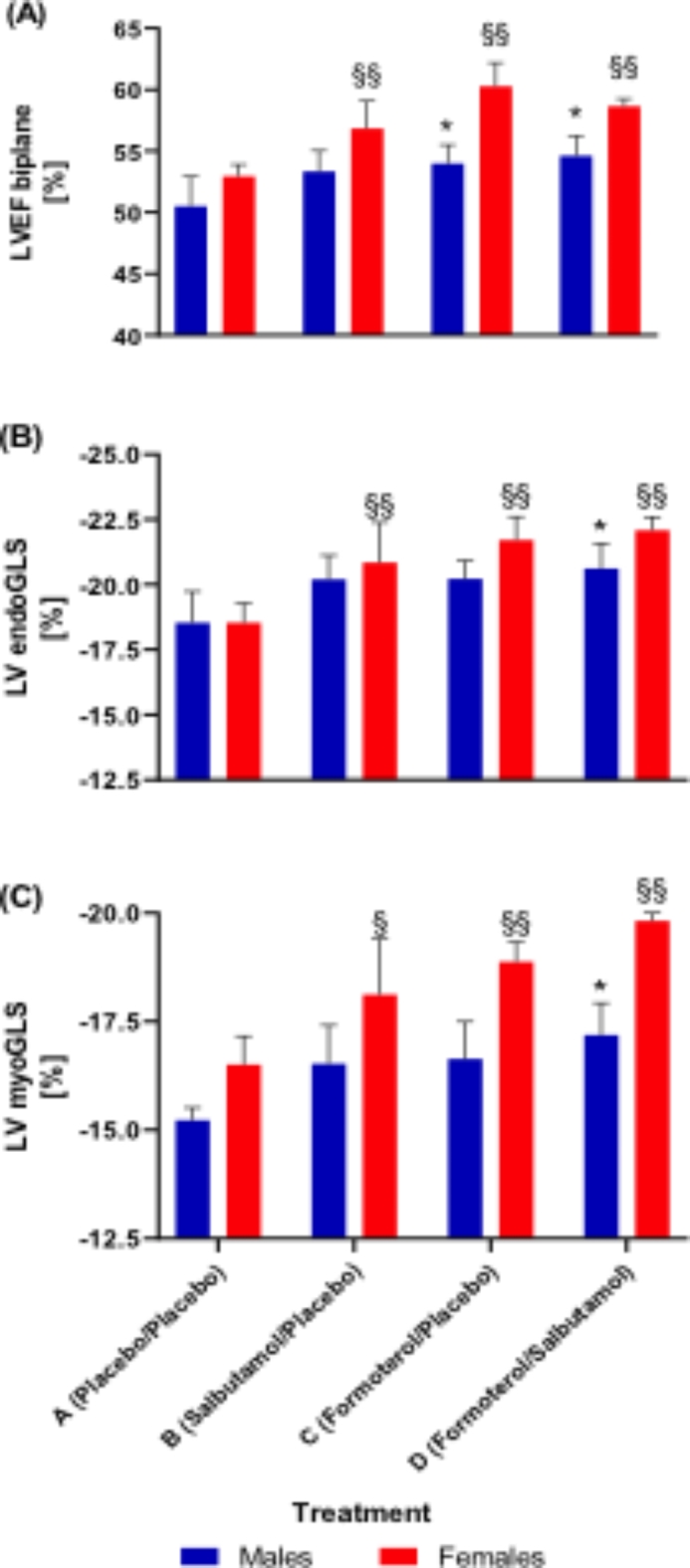
Left ventricular systolic function measured with TomTec software presented for each study arm (treatment) and sex (*n* = 12 male and 12 female participants). (**A**)LVEF biplane measurements for the whole study population (*n* = 24 participants) by treatment A: 51.74% ± 5.65%; treatment B: 55.09% ± 4.24%; treatment C: 57.13% ± 6.57%; treatment D: 56.66% ± 4.52%. (**B**) LV endoGLS measurements for the whole study population (*n* = 24 participants) by treatment A: −18.54% ± 2.78%; treatment B: −20.52% ± 3.12%; treatment C: −20.96% ± 2.76%; treatment D: −21.34% ± 2.04%. (**C**) LV myoGLS measurements for the whole study population (*n* = 24 participants) by treatment A: −15.87% ± 2.0%; treatment B: −17.32% ± 2.38%; treatment C: −17.76% ± 2.17%; treatment D: −18.53% ±. 2.06%. Data are given as mean ± standard deviation. Significance set for female at § *p* < 0.05 and §§ *p* < 0.01 for treatment A (Placebo) vs. B / C / D (Salbutamol / Formoterol / Salbutamol + Formoterol), whereas for male at ^*^p < 0.05 for treatment A vs. B / C / D, respectively. All p-values derived from linear mixed effects regression model.* LVEF biplane* left ventricular biplane ejection fraction,* LV* left ventriuclar,* endoGLS* endocardial global longitudinal strain,* myoGLS* myocardial global longitudinal strain.

In Fig. 4 the measured LVSF with TomTec software are presented for each treatment and sex. According to the applied linear mixed effects regression model the LVEF as wells as LV myocardial global longitudinal strain (myoGLS) displayed a significant effect for treatment (both *p* < 0.0001) and sex (*p* = 0.0085, *p* = 0.0004, respectively), but none for period (*p* = 0.498, *p* = 0.995, respectively) for the whole study population. In regard to LV endocardial global longitudinal strain (endoGLS), a significant treatment effect was detectable for the whole study population (*p* = 0.0001) with neither significant effect on sex (*p* = 0.265) nor on period (*p* = 0.983). The linear mixed effects regression model for LV global radial strain (GRS) was not significant for the whole study population (treatment: *p* = 0.051, sex: *p* = 0.411, period: *p* = 0.438). The model estimated changes in LVEF, endoGLS and myoGLS can be found as Supplementary Tables S2–S4 online.

### Intra- and inter-observer variability

Intra-observer analysis of LVSF variables showed good agreement between measurements for LV volume measurements, LVEF and endoGLS (LVEDV ICC 0.86 (95% CI 0.62–0.96); LVESV ICC 0.90 (95% CI 0.72–0.97); LVEF ICC 0.98 (95% CI 0.92–0.99); endoGLS ICC 0.87 (95% CI 0.63–0.96)) and poor for myoGLS (ICC 0.03; 95% CI 0.00–1.00) as well as GRS (ICC 0.23; 95% CI 0.01–0.89). Inter-observer analysis of LVSF variables showed good agreement between measurements for LVEF (ICC 0.96 (95% CI 0.86–0.99), endoGLS (ICC 0.84 (95% CI 0.57–0.95) and moderate agreement for myoGLS (ICC 0.72 (95% CI 0.36–0.92), whereas poor for GRS (ICC 0.00).

### Serum concentrations of β2 agonists and HR at predefined time points

Serum samples revealed higher concentrations of salbutamol and formoterol in females, but only salbutamol reached significance post and 3 h post TT (s. Supplementary Fig. S1A; for mean ± standard deviation and significance please s. Supplementary Table S5A), whereas formoterol concentration was significantly higher (*p* = 0.024) in females only 3 h post TT (for mean ± standard deviation and significance please s. Supplementary Table S5B and Supplementary Fig. S1B). Under combined inhalation only formoterol concentrations were significantly higher in female athletes post and 3 h post TT compared to their male counterparts (p < 0.001, respectively; for mean ± standard deviation please s. Supplementary Table S5C), whereas salbutamol concentrations did not differ significantly between the sexes at respective times measured (s. Supplementary Fig. S1C). The absolute concentrations of salbutamol and formoterol decreased gradually over the measured time points for both sexes, except for 24 h post TT, where formoterol concentration was not detectable in any serum sample. Regarding the measured HR at the predefined time points shown in Supplementary Fig. S1, one could assume an increase especially within the first 15 min after termination of the TT. However, as presented in the Supplementary Fig. S2A–C (for better visualization), there was no clear association between the measured HR and each serum concentration at the predefined time points; neither scatter plots nor Spearmans rank correlation coefficients showed a significant correlation. However, due to the very small sample size the results have to be interpreted with great caution.

### Safety events

No SAE or SUSAR occurred during the entire study and no sealed envelope had to be opened.

A laboratory finding with severe iron deficiency and hypochromic microcytic anemia (female participant) during the screening phase was classified as AE with no relationship to the medication methacholine and adjudicated as moderate/grade II according to the Common Terminology Criteria for Adverse Events (CTCAE). Since an iron substitution therapy was initiated the participant was excluded (Fig. 2). During the testing phase two AE occurred (both in female participants). One participant was treated with corticosteroids interarticular after completion of the first study arm. This finding was classified as AE with no relationship to the given medication and was adjudicated as mild/grade I according to CTCAE. However, the systemic corticosteroid application led to study exclusion and drop-out (Fig. 2; after unblinding, it turned out that the study medication administered was salbutamol). Another participant complained of headache and symptoms of cold after completing the first study arm. The complaints were classified as mild/grade I according to CTCAE and the causality was adjudicated as unlikely to the study medication. The participant could continue with the regular study protocol. The administered study medication during the first study arm was formoterol.

## Discussion

To the best of our knowledge this is the first study investigating the acute effects on LVSF of WADA-permitted iβ2A in healthy, non-asthmatic female and male endurance athletes using standard 2D TTE and STE.

The main findings in this study are (i) a significant increase in LVEF after a 10-min all out TT performance and inhalation of salbutamol and formoterol as well as after their combined inhalation for the whole study population. (ii) This significant improvement in LV contraction was also detectable for the whole study population in endoGLS as well as myoGLS. (iii) There were different effects on LVSF depending on sex: in female athletes all tested β2A significantly improved LVSF, whereas in male athletes only the combined inhalation of salbutamol plus formoterol showed a significant increase in LVSF (LVEF/endoGLS/myoGLS). (iv) No significant change in GRS was detected. (v) Significantly higher serum concentrations for all tested β2A were detectable in female athletes 3 h post TT, while higher concentrations of salbutamol where observed under the combined inhalation of salbutamol plus formoterol in male athletes post TT (but without significance).

For increasing cardiac contractility three principle mechanism are known: (i) β-adrenergic stimulation, (ii) Frank-Starling mechanism and (iii) positive force-frequency relation (“Bowditch-Treppe”)^47^. Snyder et al. had already described systemic effects with increased cardiac output and plasma norepinephrine levels after inhalation of salbutamol^35^ that might occur due to the positive inotropic effect of β2A. We assume a positive inotropic effect of the tested study medication as a cause of the increased LVSF in our healthy study population in combination with a β2-mediated peripheral vasodilation thus influencing cardiac afterload. The potential inotropic effect of iβ2A on healthy individuals has scarcely been studied and is more expected in high dose intake than in commonly prescribed doses for anti-asthmatic β2-agonist therapy^48^. Nevertheless, the most reported adverse side effects are tachycardia, supraventricular and ventricular arrhythmias, hyopkalemia, gastrointestinal disturbances or tremor. They may appear when β2A are overdosed/misused^11,49^ and there are also reports of fatalities (amongst others myocardial ischemia and cardiac arrest) when misusing β2A^49–51^.

In our study population, there were no side effects related to the study medication, although the used doses were higher than the usual prescribed for pulmonary patients (for salbutamol an acute cumulative dose of 1200 µg was applied and for formoterol 36 µg, respectively). This may be due to the fact that the doses administered were still lower than the current WADA-allowed thresholds^24^. The observed higher serum β2A concentrations in our female participants might be the reason for the increase of LVSF most likely due to β-adrenergic stimulation. The higher concentration of salbutamol after the combined inhalation of salbutamol + formoterol in male athletes (compared to their female counterparts) might therefore also result in an enhanced β-adrenergic stimulation explaining the detected significant increase in LVSF. One previous study reported a significant increase in LVEF (measured by multiple gated radionuclide ventriculography) in patients with chronic bronchitis after oral intake of salbutamol (after 60 and 90 min) and a variable response after inhalation of 5 mg salbutamol with a trend towards an increase in LVEF^52^. These changes were most likely to be caused by a reduction in systemic vascular resistance rather than a direct inotropic effect^52^. One recently published, double-blind, randomized, crossover, placebo-controlled single-center study examined the effect of a combination therapy of a long-acting β2A (indacaterol) and muscarinic antagonist (glycopyrronium) on cardiac function in patients with chronic obstructive pulmonary disease and lung hyperinflation^53^. Here, dual bronchodilator not only improved lung function and deflation, but also significantly increased LV end-diastolic volume (EDV) measured by cardiac magnetic resonance imaging, with improved cardiac index mediated by increased stroke volume^53^. Mechanistically, the increase in LV EDV might occur due to lung deflation which increases left and right ventricular preload with an optimized biventricular filling and cardiac index according to Frank-Starling mechanism^53^. In addition, to pulmonary volume and vascular changes caused by dual bronchodilation may also affect ventricular preload suggesting that the detected cardiac changes are not due to a direct inotropic or chronotropic effect, since patients receiving β-blockers displayed similar effects and no changes in HR with the study treatment (taken once daily for 14 days)^53^. Similarly, we found no correlation between serum concentration and measured HR at the predefined time points. The only measurable difference for the significant changes in LVSF seems to be the higher serum concentration of the tested β2A. Nevertheless, the observed improvement in LVSF with administration of β2A did not result in significant changes in TT performance although significant changes in lung function were detected as previously reported by Bizjak et al.^13^. A β2-mediated respiratory-centered peripheral vasodilation could additionally influence performance and/or cardiac function; other influencing factors could have been differences in lung diffusing capacity for carbon monoxide (DLCO), pulmonary capillary blood volume (Vc) and diffusing membrane capacity (DM). An increase in DLCO, DM and Vc has been described in endurance-trained as well as non-endurance-trained athletes with increasing exercise intensity^54^. At 80–90% of V̇O_2max_ endurance-trained athletes had higher DLCO and greater DM, but no differences in Vc during exercise, which might explain for differences within the pulmonary membrane facilitating the increased oxygen demand^54^. In contrast, another study group found neither an increase in DLCO nor in DM after application of nebulized albuterol in healthy humans^55^. Lazovic et al. found no influence on spirometry indices and DLCO by either aerobic or anaerobic training type^56^. Even in patients with chronic obstructive pulmonary disease, significant improvements in lung function, including DLCO at rest, were detected after inhalation of terbutaline, but without significant improvement in exercise capacity^57^. With reference to the literature presented, a potential impact of iβ2A and/or exercise capacity on these parameters was not really expected, and therefore we did not investigate these possible interferences as the main focus was on cardiac function.

We performed the echocardiographic quantification of LVSF at the latest 15 min after termination of each TT, which was approximately 45 min after inhalation of the study medication (that was only once administered, but higher than the usual recommended single dosage; s. Fig. 1). Therefore, the HR during echocardiographic image acquisition could have been slightly higher than the usual average resting HR what might affect the LVSF. Erbel et al. analyzed the influence of HR during atrial pacing on LVEF (measured by 2D TTE using a disc method as well as by cineventriculography) in normal control subjects and observed that an increase in HR of 10 bpm caused a decrease in LVEF by 1%^58^. Moreover, Gruca et al. reported a correlation of lower HR at rest as well as after maximal exertion and increase in GLS^59^. Therefore, our detected increase in LVSF after inhalation of study medication cannot be explained by a slightly higher HR after the acute bout of exercise alone, especially since no improvement in LVSF under placebo was detectable. Whether different preload settings may have influenced the observed changes in LVSF in our study population cannot be excluded with certainty. Nevertheless, all participants were fasting at least 8 h prior to each exercise testing and were provided with a defined and standardized breakfast (in order to standardize nutritional status and drinking quantity; for further information on standardization please see Fig. 1 and published study protocol^42^) so that the preload setting might at least be better comparable.

GLS is a sensitive and robust index^44^ and currently recommended in cardio-oncology guidelines as additional routine echocardiographic parameter for cardiovascular monitoring during cancer therapy^60–62^. To date only data on changes in LV strain after exercise in healthy subjects and/or athletes are published but no data on changes in GLS after inhalation of β2A in healthy subjects and/or athletes are yet available. Gruca et al. examined 111 elite basketball athletes (all African American) before and after completing a treadmill stress test to maximal exertion or completion of Bruce protocol and detected an increase in mean GLS magnitude^59^. Moreover, lower resting HR and lower HR at peak exercise correlated with an increase in GLS from exercise^59^. However, one third of the examined elite basketball players did not increase their GLS in response to exercise^59^. Whether this might have prognostic implication for the athletes is yet not known. Almost all scientific evidence on the prognostic validity of GLS is commonly related to reduced and not-improving/increasing GLS, yet. However, increases in GLS have already been observed in dobutamine stress echocardiography that correlated with clinical prognosis, but no reference values for GLS augmentation are yet established^63,64^.

Nevertheless, our data indicate a significant effect of iβ2A on LV contractility in healthy endurance athletes, which should be more closely investigated in further studies. Moreover, a sex effect of the used β2A was detectable on LVSF as well as sex-dependent significantly higher serum β2A concentrations. In female athletes the treatment effect on LVSF after inhalation of β2A in WADA-permitted dosage and TT performance was more pronounced than in male athletes. In men, only the combined inhalation of salbutamol plus formoterol showed a significant change in LVSF. This could be due to the fact that female participants had lower body weight combined with a higher body fat mass. This results in a significantly lower fat-free mass, i.e., a significantly reduced volume of distribution for hydrophilic drugs. This might also explain why all measured absolute concentrations of β2A were higher in female athletes compared to males (except for the combined administration, where salbutamol concentration post TT was non-significantly higher in men). This sex difference may also be due to faster distribution in blood and longer systemic circulation. Thus, it can be assumed that higher serum levels of β2A are achieved on average in female subjects despite smaller lung volumes/capacity and that women could possibly benefit from sex-unspecific maximum doses in sports. Sex-specific changes in body composition had recently been reported in well-trained female athletes in conjunction with a period of aerobic training and inhaled salbutamol^8^. Hostrup et al. even detected a temporarily lower increase in V̇O_2_max in salbutamol-treated vs. placebo-treated participants during the first 4 weeks of training intervention and a marked decline in isometric muscle torque, but both sex-unspecific^8^.

In summary, out data indicate a significant improvement in LVSF after inhalation of β2A in WADA-permitted dosage and TT performance, which is less pronounced in male athletes. In this context, the inhalation of β2A in WADA-permitted dosage seems to increase cardiac contractility in healthy endurance athletes - predominantly in women.

## Limitations

This study was a single-center study with a small number of participants who were all Caucasian and healthy, which might limit general and clinical translation to other study populations. In addition, β2A were taken once at the predefined dosage as “a single” acute treatment in healthy volunteers rather than regularly as in chronic asthmatic athletes, which might have different effects and/or additive effects as previously reported^8^. Our standard protocol for performance testing with a 10-minute all-out TT at 90–95% of the respective power output at V̇O_2_max does not represent realistic conditions e.g. in professional road racing, as time trial competitions usually have a much longer duration between 30 and 50 min with a resulting lower cardiorespiratory and a slightly lower power output due to the longer duration. Moreover, the echocardiographic examinations were performed 15 min after TT performance and not immediately after the acute bout of exercise that may have shown different results as potential effects may be only short-timed.

## Conclusion

The single inhalation of salbutamol and formoterol in therapeutically used doses (below WADA-threshold) lead to elevated serum concentrations and improved LVSF in healthy endurance athletes. These effects were increased by the combined application of both drugs and were more pronounced in female athletes indicating both a dose-dependent and a sex effect on cardiac function. Although no adverse cardiac side effects occurred during the study, the finding may indicate potential risk when increasing dose above the tested concentrations, particular in women with smaller body weight and plasma volume. These findings warrant further examination.

## Electronic supplementary material

Below is the link to the electronic supplementary material.

Supplementary Material 1

Supplementary Material 2

Supplementary Material 3

Supplementary Material 4

Supplementary Material 5

Supplementary Material 6

## Data Availability

The detailed data of all biometric analysis results are provided in the data repository OPARU of Ulm University under doi: 10.18725/OPARU-47213^65^ and directly under the following link: https://oparu.uni-ulm.de/items/d49fecee-0aac-47ed-9060-7703a829b76a.

## Abbreviations

CPX: Cardiopulmonary exercise test
ECG: Electrocardiogram
HR: Heart rate
iβ2A: Inhaled beta-2 adrenoceptor agonists
LAD: Lower airway disease/dysfunction
LVSF: Left ventricular systolic function
TT: Time trial
TTE: Transthoracic echocardiography
V̇O_2_max: Maximum oxygen uptake
WADA: World anti-doping agency
