# Supplementary material for: Left ventricular systolic function after inhalation of beta-2 agonists in healthy athletes
Source: Sci Rep. 2024 Oct 8;14:23437. doi: 10.1038/s41598-024-74095-z (PMC11461498; doi:10.1038/s41598-024-74095-z)
Supplement: Supplementary file 1 — Supplementary Material 1 [file 41598_2024_74095_MOESM1_ESM.docx]

**Supplementary Table S1: Measured variables from pulmonary function tests performed during the screening phase**

| Values | Female (n=12) | | Male (n=12) | |
| --- | --- | --- | --- | --- |
|  | Mean ± SD | % predicted | Mean ± SD | % predicted |
| FVC (L) | 4.578 ± 0.56 | 106.3 ± 13,9 | 6.101 ± 0.6 | 107.1 ±9.2 |
| FEV1 (L) | 3.716 ± 0.54 | 100.8 ± 16.6 | 4.835 ± 0.59 | 101.6 ± 10.4 |
| MEF25 (L/s) | 1.918 ±0.59 | 94.8 ± 32.99 | 2.278 ±0.65 | 99.9 ± 26.6 |
| TLC (L)# | 5.6 ± 0.53 | 103.3 ± 7.75 | 7.52 ± 0.93 | 104.9 ± 11.9 |
| FRC (L)# | 2.72 ± 0.48 | 95.7 ± 14.8 | 3.30 ± 0.72 | 99.5 ± 22.1 |
| RV (L) # | 1.03 ± 0.32 | 69.4 ±20.5 | 1.47 ± 0.37 | 87.3 ± 21.2 |
| sRaw (kPa*s) # | 0.81 ± 0.2 | NA | 1.01 ± 0.32 | NA |

Data are given as mean ± standard deviation or absolute numbers, unless otherwise stated.

Pulmonary function test included both spirometry (Qark SPIRO, COSMED, Rome, Italy) and whole-body plethysmography (Q Box, COSMED, Rome, Italy). FVC: forced vital capacity; FEV1: forced expiratory volume in 1 s; MEF25: mean expiratory flow at 25% of forced vital capacity; TLC: total lung capacity; FRC: functional residual capacity; RV: residual volume; sRaw: specific airway resistance; NA: not applicable.

**Supplementary Table S2: Linear mixed effects regression model for EF biplan in % with differences of least squares means**

| Whole Study Population (n = 24) | | | |
| --- | --- | --- | --- |
| Treat | Estimate | 95% CI (lower; upper) | p-value |
| A vs. B | -3.37 | -5.58; -1.16 | 0.0034 |
| A vs. C | -5.40 | -7.61; -3.19 | <.0001 |
| A vs. D | -4.83 | -7.07; -2.59 | <.0001 |
| B vs. C | -2.04 | -4.25; 0.18 | 0.0705 |
| B vs. D | -1.47 | -3.71; 0.77 | 0.1958 |
| C vs. D | 0.57 | -1.67; 2.81 | 0.6142 |
|  |  |  |  |
| Female only (n = 12) | | | |
| A vs. B | -3.9 | -6.57; -1.24 | 0.0055 |
| A vs. C | -7.34 | -10.00; -4.67 | <.0001 |
| A vs. D | -5.71 | -8.39; -3.05 | 0.0001 |
| B vs. C | -3.43 | -6.10; -0.77 | 0.0134 |
| B vs. D | -1.81 | -4.48; 0.86 | 0.1760 |
| C vs. D | 1.62 | -1.04; 4.29 | 0.2235 |
|  |  |  |  |
| Male only (n = 12) | | | |
| A vs. B | -2.83 | -6.17; 0.52 | 0.0944 |
| A vs. C | -3.47 | -6.81; -0.12 | 0.0427 |
| A vs. D | -3.77 | -7.22; -0.33 | 0.0330 |
| B vs. C | -0.64 | -3.98; 2.71 | 0.6992 |
| B vs. D | -0.94 | -4.39; 2,50 | 0.5795 |
| C vs. D | -0.31 | -3.75; 3.14 | 0.8572 |

EF: ejection fraction; Treat A: placebo/placebo; Treat B: salbumatol/placebo; Treat C: formoterol/placebo; Treat D: salbutamol/formoterol; CI: confidence interval

**Supplementary Table S3: Linear mixed effects regression model for endoGLS in % with differences of least squares means**

| Whole Study Population (n = 24) | | | |
| --- | --- | --- | --- |
| Treat | Estimate | 95% CI (lower; upper) | p-value |
| A vs. B | 1.98 | 0.74; 3.23 | 0.0022 |
| A vs. C | 2.42 | 1.18; 3.67 | 0.0002 |
| A vs. D | 2.80 | 1.53; 4.06 | <.0001 |
| B vs. C | 0.44 | -0.81; 1.69 | 0.4833 |
| B vs. D | 0.81 | -0.45; 2.07 | 0.2038 |
| C vs. D | 0.37 | -0.89; 1.63 | 0.5586 |
|  |  |  |  |
| Female only (n = 12) | | | |
| A vs. B | 2.31 | 0.60; 4.02 | 0.0100 |
| A vs. C | 3.17 | 1.46; 4.89 | 0.0007 |
| A vs. D | 3.55 | 1.84; 5.27 | 0.0002 |
| B vs. C | 0.86 | -0.85; 2.58 | 0.3127 |
| B vs. D | 1.24 | -0.47; 2.96 | 0.1494 |
| C vs. D | 0.38 | -1.33; 2.09 | 0.6539 |
|  |  |  |  |
| Male only (n = 12) | | | |
| A vs. B | 1.66 | -0.17; 3.49 | 0.0735 |
| A vs. C | 1.68 | -0.15; 3.50 | 0.0707 |
| A vs. D | 1.91 | 0.02; 3.79 | 0.0473 |
| B vs. C | 0.02 | -1.81; 1.85 | 0.9845 |
| B vs. D | 0.25 | -1.64; 2.13 | 0.7889 |
| C vs. D | 0.23 | -1.65; 2.12 | 0.8035 |

Endo: endocardial; GLS: global longitudinal strain; Treat A: placebo/placebo; Treat B: salbumatol/placebo; Treat C: formoterol/placebo; Treat D: salbutamol/formoterol; CI: confidence interval

**Supplementary Table S4: Linear mixed effects regression model for myoGLS in % with differences of least squares means**

| Whole Study Population (n = 24) | | | |
| --- | --- | --- | --- |
| Treat | Estimate | 95% CI (lower; uper) | p-value |
| A vs. B | 1.46 | 0.45; 2.46 | 0.0052 |
| A vs. C | 1.89 | 0.89; 2.90 | 0.0004 |
| A vs. D | 2.64 | 1.63; 3.66 | <.0001 |
| B vs. C | 0.44 | -0.57; 1.44 | 0.3884 |
| B vs. D | 1.19 | 0.17; 2.21 | 0.0227 |
| C vs. D | 0.75 | 0.27; 1.77 | 0.1446 |
|  |  |  |  |
| Female only (n = 12) | | | |
| A vs. B | 1.62 | 0.19; 3.05 | 0.0283 |
| A vs. C | 2.38 | 0.94; 3.81 | 0.0020 |
| A vs. D | 3.31 | 1.88; 4.75 | <.0001 |
| B vs. C | 0.76 | -0.68; 2.19 | 0.2887 |
| B vs. D | 1.69 | 0.26; 3.13 | 0.0222 |
| C vs. D | 0.93 | -0.50; 2.37 | 0.1933 |
|  |  |  |  |
| Male only (n = 12) | | | |
| A vs. B | 1.29 | -0.16; 2.74 | 0.0782 |
| A vs. C | 1.41 | -0.04; 2.86 | 0.0563 |
| A vs. D | 1.89 | 0.40; 3.38 | 0.0147 |
| B vs. C | 0.12 | -1.33; 1.56 | 0.8721 |
| B vs. D | 0.60 | -0.89; 2.09 | 0.4188 |
| C vs. D | 0.48 | -1.01; 1.97 | 0.5129 |

Myo: myocardial; GLS: global longitudinal strain; Treat A: placebo/placebo; Treat B: salbumatol/placebo; Treat C: formoterol/placebo; Treat D: salbutamol/formoterol; CI: confidence interval

**Supplementary Table S5A: Measured serum concentrations after the inhalation of 1200 µg salbutamol measured by UHPLC-MS/MS at predefined time points**

|  | Whole study group (n=24) | Female (n=12) | Male (n=12) |
| --- | --- | --- | --- |
| Pre (ng/ml) | ND | ND | ND |
| Post (ng/ml) | 2.36 ± 1.06 | 2.89 ± 0.84** | 1.83 ± 1.01** |
| Post 3 h (ng/ml) | 1.63 ± 0.70 | 2.04 ± 0.47* | 1.23 ± 0.67* |
| Post 24 h (ng/ml) | 0.18 ± 0.11 | 0.19 ± 0.09 | 0.16 ± 0.12 |

Data are given as mean ± standard deviation, unless otherwise stated.

Significance set at * p<0.05 and **p<0.001 between male and female participants.

UHPLC-MS/MS: ultra-high-performance liquid chromatography hyphenated to tandem mass sepctrometry; Pre: serum concentration before the load; Post: serum concentration after the load; Post 3 h : serum concentration 3 hours after the load; Post 24 h: serum concentration 24 hours after the load

**Supplementary Table S5B: Measured serum concentrations after the inhalation of 36 µg formoterol measured by UHPLC-MS/MS at predefined time points**

|  | Whole study group (n=24) | Female (n=12) | Male (n=12) |
| --- | --- | --- | --- |
| Pre (ng/ml) | ND | ND | ND |
| Post (ng/ml) | 0.004 ± 0.003 | 0.005 ± 0.004 | 0.003 ± 0.002 |
| Post 3 h (ng/ml) | 0.003 ± 0.003 | 0.004 ± 0.003* | 0.002 ± 0.002* |
| Post 24 h (ng/ml) | ND | ND | ND |

Data are given as mean ± standard deviation, unless otherwise stated.

Significance set at * p<0.05 between male and female participants.

UHPLC-MS/MS: ultra-high-performance liquid chromatography hyphenated to tandem mass sepctrometry; Pre: serum concentration before the load; Post: serum concentration after the load; Post 3 h : serum concentration 3 hours after the load; Post 24 h: serum concentration 24 hours after the load

**Supplementary Table S5C: Measured serum concentrations after the combined inhalation of 1200 µg salbutamol and 36 µg formoterol measured by UHPLC-MS/MS at predefined time points**

|  | Whole study group (n=24) | Female (n=12) | Male (n=12) |
| --- | --- | --- | --- |
| Measured serum concentration after inhalation of 1200 µg salbutamol | | | |
| Pre (ng/ml) | ND | ND | ND |
| Post (ng/ml) | 2.36 ± 1.06 | 2.26 ± 1.06 | 3.02 ±3.05 |
| Post 3 h (ng/ml) | 1.53 ± 0.59 | 1.72 ± 0.53 | 1.34 ± 0.60 |
| Post 24 h (ng/ml) | 0.17 ± 0.07 | 0.10 ± 0.07 | 0.16 ± 0.06 |
| Measured serum concentration after inhalation of 36 µg formoterol | | | |
| Pre (ng/ml) | ND | ND | ND |
| Post (ng/ml) | 0.005 ± 0.004 | 0.008 ± 0.004** | 0.003 ± 0.003** |
| Post 3 h(ng/ml) | 0.004 ± 0.003 | 0.006 ± 0.003** | 0.002 ± 0.002** |
| Post 24 h (ng/ml) | ND | ND | ND |

Data are given as mean ± standard deviation, unless otherwise stated.

Significance set at ** p<0.001 between male and female participants.

UHPLC-MS/MS: ultra-high-performance liquid chromatography hyphenated to tandem mass sepctrometry; Pre: serum concentration before the load; Post: serum concentration after the load; Post 3 h : serum concentration 3 hours after the load; Post 24 h: serum concentration 24 hours after the load

***Supplementary Figure Legend***

*Supplementary Figure S1:*

Serum concentrations after inhalation of 1200 µg salbutamol (A), 36 µg formoterol (B) and their combined application (C) measured by UHPLC-MS/MS and *measured HR* presented for the whole study group (n=24) as well as for each sex (n=12 male and 12 female participants) *at predefined time points;* modified from Bizjak et al.^13^*.*

*Data for serum concentration are presented as violitin blots. The width of each curve corresponds with the approximate frequency of data points in each region. For the mean ± standard deviation please see supplementary tables S3A-S3C. Data for HR are presented as measured values in bpm at the predefined time points.*

*UHPLC-MS/MS: ultra-high-performance liquid chromatography hyphenated to tandem mass sepctrometry; HR: hear rate measured with a heart rate monitor; pre: serum concentration or HR before the load; post: serum concentration or HR after the load; post 3h : serum concentration or HR 3 hours after the load; post 24 h: serum concentration or HR 24 hours after the load.*

*Supplementary Figure S2:*

*Correlation between the measured HR and serum concentration after inhalation of 1200 µg salbutamol (S2A), 36 µg formoterol (S2B) and their combined application (S2C) measured by UHPLC-MS/MS and presented for the whole study group (n=24) as well as for each sex (n=12 male and 12 female participants) at predefined time points.*

*Data are presented as measured values for HR in bpm and for serum concentration of salbutamol, formotoeral and their combined application in ng/ml, resprectively.*

*Black rings represent the whole study group (n=24), the red and the blue rings represent female (n=12) and male (n=12) particapants respectively.*

*No association between HR and each serum concentration was detected using scatter plots and Spearmans rank correlation coefficients.*

*HR: heart rate; UHPLC-MS/MS: ultra-high-performance liquid chromatography hyphenated to tandem mass sepctrometry; pre: HR and serum concentration before the load; post: HR and serum concentration after the load; post 3 h: HR and serum concentration 3 hours after the load; post 24 h: HR and serum concentration 24 hours after the load; bpm: beats per minute.*
