# Supplementary figures and images for: Left ventricular systolic function after inhalation of beta-2 agonists in healthy athletes
Source: Sci Rep. 2024 Oct 8;14:23437. doi: 10.1038/s41598-024-74095-z (PMC11461498; doi:10.1038/s41598-024-74095-z)

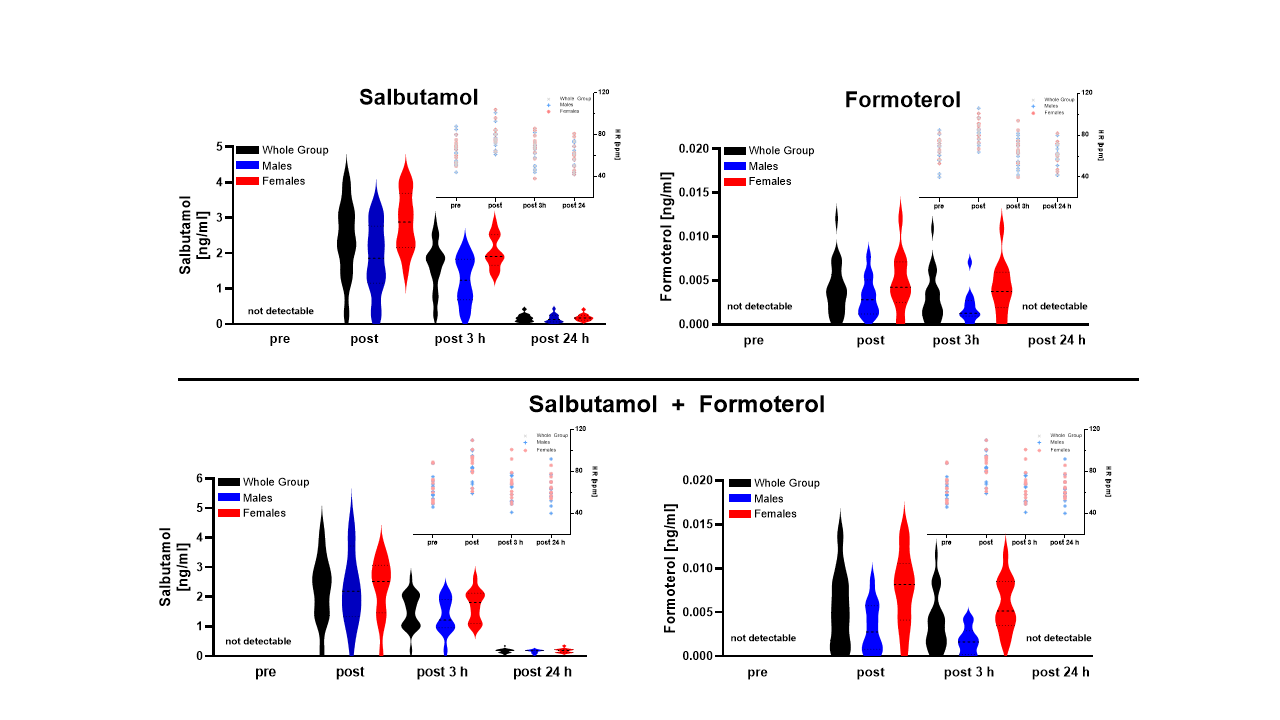

Supplement: Supplementary file 2 — Supplementary Material 2 [file 41598_2024_74095_MOESM2_ESM.tif]

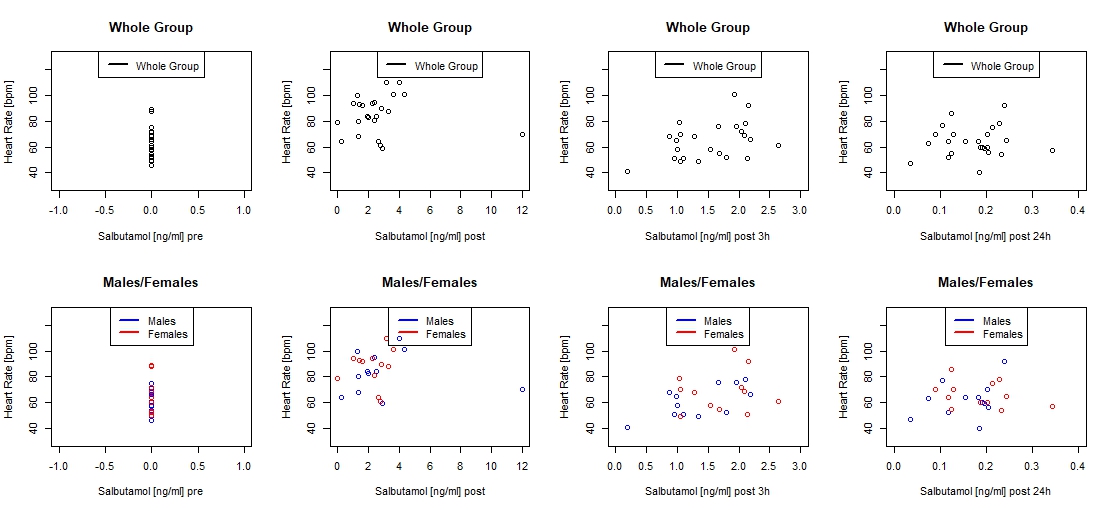

Supplement: Supplementary file 3 — Supplementary Material 3 [file 41598_2024_74095_MOESM3_ESM.jpeg]

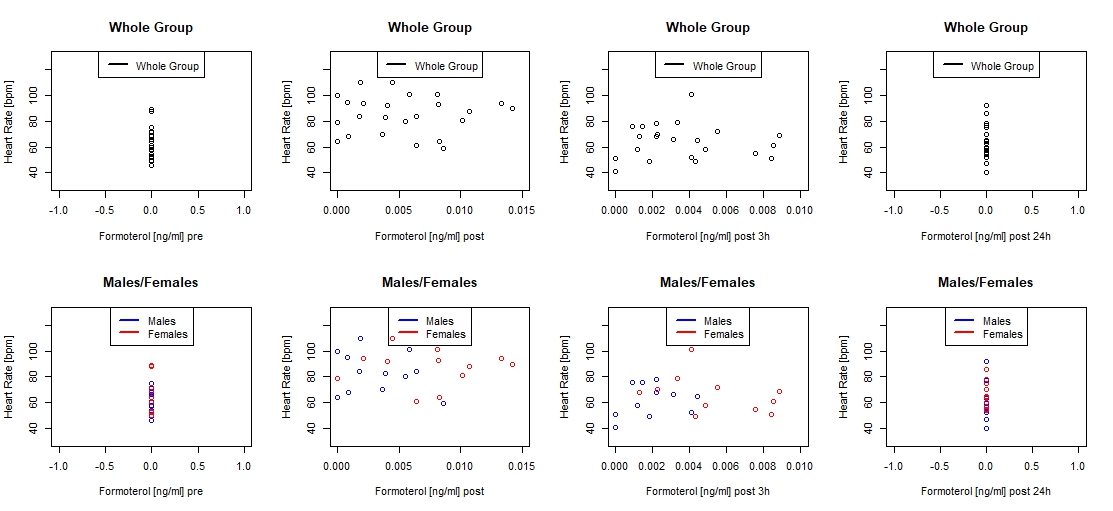

Supplement: Supplementary file 4 — Supplementary Material 4 [file 41598_2024_74095_MOESM4_ESM.jpeg]

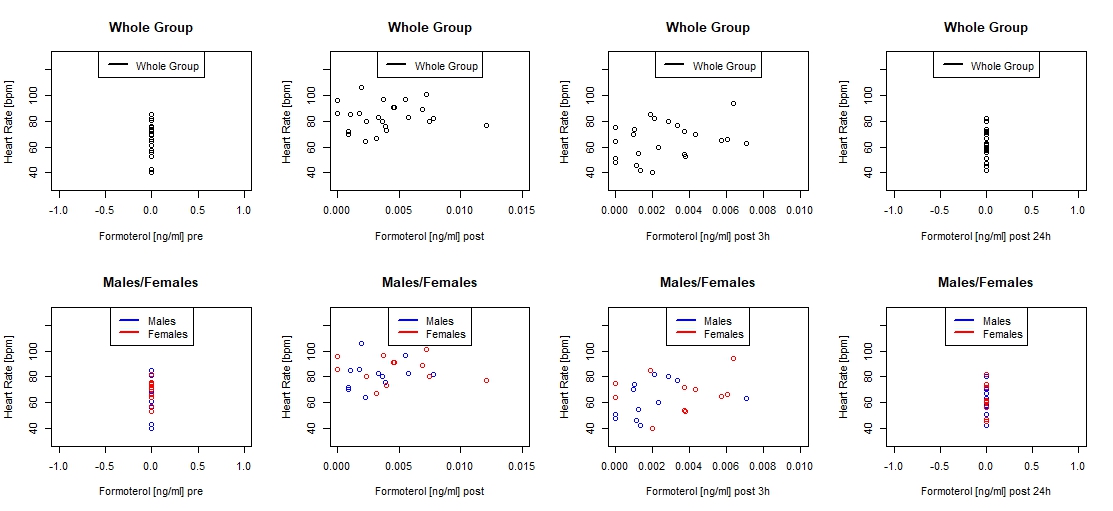

Supplement: Supplementary file 5 — Supplementary Material 5 [file 41598_2024_74095_MOESM5_ESM.jpeg]

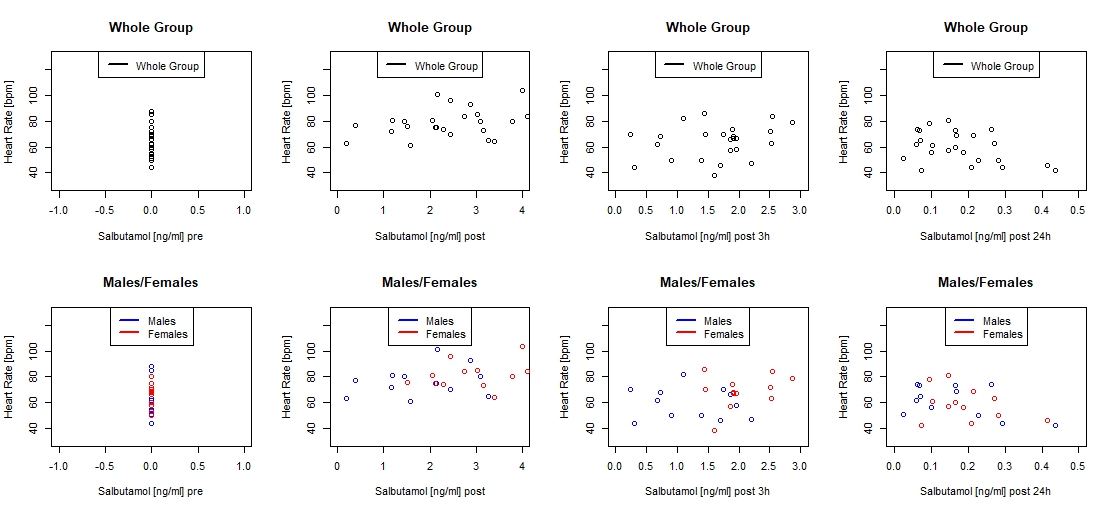

Supplement: Supplementary file 6 — Supplementary Material 6 [file 41598_2024_74095_MOESM6_ESM.jpeg]
